# The efficacy and safety of Longmu Tang granule for the treatment of atopic dermatitis: study protocol for a single-centred, double-blinded, randomised, placebo-controlled trial

**DOI:** 10.1186/s13063-022-06313-w

**Published:** 2022-05-09

**Authors:** Ya-qin Li, Tao-tao Shen, Qing-ying Wang, Meng-xi Ma, Feng-yan Tian, Yuan-yao She, Yi-cheng Tao, Jing-jing Wang, Hui-yan Chi, Na Lang, Jian-xun Ren

**Affiliations:** Department of Dermatology, Xiyuan Hospital of China Academy of Chinese Medical Sciences, Beijing, 100091 China

**Keywords:** Atopic dermatitis, Anti-inflammation, Longmu Tang granule, Randomised controlled trial, Traditional Chinese medicine

## Abstract

**Background:**

Atopic dermatitis (AD) is a chronic relapsing skin disease that has long-term physical and mental health impacts on children with this condition. Current treatments mainly include anti-inflammatory, antibacterial, and anti-allergic interventions, systemic therapy, and recently emerging target-focused agents. However, these treatments have limited effectiveness and unwanted side effects. The use of traditional Chinese medicine (TCM) in the treatment of AD has a long history, with promising efficacies, low toxicity, and improvements in the quality of life of patients with AD. Longmu Tang granule, a TCM, has been used to effectively treat AD since 2008 through doctors’ prescriptions. To scientifically evaluate the clinical efficacy and safety of Longmu Tang granule, we proposed to launch a single-centred, double-blinded, randomised, placebo-controlled trial.

**Methods:**

In this single-centred, double-blinded, randomised, placebo-controlled clinical trial conducted at Xiyuan Hospital of China Academy of Chinese Medical Sciences, a total of 60 participants will be randomly assigned (1:1) to receive the Longmu Tang granule or placebo granule for 8 weeks. The primary outcome will be evaluated using the index of Scoring Atopic Dermatitis. The secondary outcomes will be evaluated using the Children’s Dermatology Life Quality Index and the number cancellation test. The mechanistic evidence will be the serum levels of inflammatory cytokines, including immunoglobulin E, tumour necrosis factor-α, interleukin-1, and interleukin-6.

**Discussion:**

The results of this trial will provide evidence of the efficacy and safety of the Longmu Tang granule and prove its anti-inflammatory action in patients with AD.

**Trial registration:**

Chinese Clinical Trial Registry Chictr.org ID: ChiCTR2100041591. Registered on 1 January 2021

## Administrative information

Note: the numbers in curly brackets in this protocol refer to SPIRIT checklist item numbers. The order of the items has been modified to group similar items (see http://www.equator-network.org/reporting-guidelines/spirit-2013-statement-defining-standard-protocol-items-for-clinical-trials/).

**Table:** 

Title {1}	The efficacy and safety of Longmu Tang granule for the treatment of atopic dermatitis: study protocol for a single-centred, double-blinded, randomised, placebo-controlled trial
Trial registration {2a and 2b}.	Chictr.org ID: ChiCTR2100041591. Registered on 1 January 2021.
Protocol version {3}	14, May 2020, version 3.2
Funding {4}	This work is supported by Chinese Capital’s Funds for Health Improvement and Research (number: 2020-2-4172)
Author details {5a}	Department of Dermatology, Xiyuan Hospital of China Academy of Chinese Medical Sciences, Beijing 100091, China
Name and contact information for the trial sponsor {5b}	Contact number: 8610-88549772
Role of sponsor {5c}	Capital’s Funds for Health Improvement and Research will provide the funding for this trial and supervise its progress

## Introduction

### Background and rationale {6a}

Atopic dermatitis (AD) is one of the most common chronic relapsing skin diseases and is characterised by severe itching, papules, dryness, exudation, and scarring in infants, children, and adults [1]. A recent epidemiological study showed that the prevalence of AD was as high as 10–20% in developed countries [2]. AD affects up to 12.94% of children aged 1–7 years in 12 cities in China [3]. AD is not only associated with food allergies, asthma, allergic rhinitis, and other immune-mediated inflammatory diseases; recent literature also suggests AD is associated with attention-deficit/hyperactivity disorder (ADHD) and mental health disorders, such as depression [4–7]. The typical clinical features of AD are intensive pruritic and eczematous dermatitis that can severely affect quality of life. AD has a greater impact on children's quality of life than other chronic childhood diseases, including asthma and type 1 diabetes [8]. Families and caregivers experience significant stress and high expenditures because of difficult and time-consuming treatments [9]. Thus, AD is associated with substantial distress, reduced quality of life, and increased resource demands for families.

Although the underlying pathogenesis of AD is not fully understood, it is known to be caused by complex interactions of abnormal barrier function, cutaneous microbiome invasion, and predominantly type-2-skewed immune dysregulation [10]. AD lesions show destruction of the skin barrier caused by inflammatory cytokines from T helper 2 cell-mediated immunity. Patients with mild-to-moderate AD develop red, oozing, and crusted rashes, which have chronic recurrence and may be limited to the face, scalp, hands, arms, feet, or legs. Pruritus is the major subjective symptom, and it causes frequent scratching, which may exacerbate the itch-and-scratch cycle and result in secondary skin infections. Pruritus and frequent scratching at night cause difficulty in falling asleep and affect the quality of life. Lack of sleep at night and early waking can induce adverse consequences in children with AD, such as psychological disorders, inattention, behavioural changes, and difficulty in remembering daily affairs. Thus, there is a need to search for more effective novel therapeutic strategies to alleviate chronic inflammation, including antibacterial and anti-allergic effects, reduce chronic itching, and improve quality of life in the long-term management of AD.

Traditional Chinese medicine (TCM) has shown multitargeted effectiveness with a satisfactory safety profile in improving the symptom severity and quality of life of patients with AD, especially for those with refractory AD [11, 12]. According to TCM theory, damp-heat disturbing heart-mind is the most common syndrome in the pathogenesis of AD and is characterised by fatigue, shortness of breath, spontaneous perspiration or night sweating palpitation, dry mouth, attention deficits, extroversion, restlessness, insomnia, and other symptoms. Longmu Tang is composed of Os Draconis (Long Gu; 30 g/126 g), Concha Ostreae (Calcined Mu Li; 30 g/126 g), Forsythia suspensa (Lian Qiao; 15 g/126 g), Massa Medicata Fermentata (Toasted Shen Qu; 15 g/126 g), Poria cocos (Fu Ling Pi; 30 g/126 g), and Radixet Rhizonma Ephedrae (Ma Huang Gen; 6 g/126 g) (Table 1). Studies have revealed the following. (1) Os Draconis and Concha Ostreae, the key components of Longmu Tang, exert sedation-like and antipruritic effects in the treatment of AD. In addition, organic calcium in Os Draconis and Concha Ostreae can alleviate allergic symptoms [13]. (2) Forsythia suspensa inhibits *Staphylococcus aureus* on the skin surface of patients with AD [14, 15]; and (3) Poria cocos bark extract has potential as an oral immune suppressor for the treatment of AD through the generation of regulatory T cells [16]. In a previous trial, we found that the Longmu Tang granule could inhibit skin lesions and attenuate atopic itch through suppression of inflammation in AD [17]. We intend to conduct a high-quality, well-designed, randomised, placebo-controlled, double-blind clinical trial to further confirm the efficacy and safety of the Longmu Tang granule for the treatment of patients with mild-to-moderate AD.

**Table 1:** Components of Longmu Tang granule

Chinese name	Latin name	English name
Long Gu	Os Draconis	Fossilizid
Calcined Mu Li	Concha Ostreae	Calcined concha ostreae
Lian Qiao	Forsythia suspensa	Fructus forsythiae
Toasted Shen Qu	Massa Medicata Fermentata	Toasted medicated leaven
Fu Ling Pi	Poria cocos	Tuckahoe peel
Ma Huang Gen	Radixet Rhizonma Ephedrae	Radix ephedrae

### Objectives {7}

#### Aims

The aim of this randomised controlled trial will be to evaluate the effects and safety of the Longmu Tang granule in the treatment of AD.

#### Hypothesis

This study hypothesised that the Longmu Tang granule will be able to improve the clinical symptoms, quality of life, and cognitive ability of patients with mild or moderate AD by attenuating inflammation in comparison with the placebo.

#### Objectives

1. To evaluate the efficacy of Longmu Tang granule in the treatment of AD
2. To determine whether Longmu Tang granule will improve the quality of life of patients with AD
3. To determine whether Longmu Tang granule will improve the cognitive ability of patients with AD
4. To explore the mechanism of Longmu Tang granule in the treatment of AD

### Trial design {8}

This study will be a single-centred, double-blinded, randomised, placebo-controlled clinical trial. The aim of this study is to assess the safety and effectiveness of Longmu Tang granule in treating AD with damp-heat disturbing heart-mind. The trial was registered in the Chinese Clinical Trial Registry (ChiCTR.org ID: ChiCTR2100041591).

## Methods: participants, interventions and outcomes

### Study setting {9}

This trial will be conducted at Xiyuan Hospital of China Academy of Chinese Medical Sciences. Sixty patients will be recruited for this study. All patients will be informed about the objective, approaches, potential adverse effects, and advantages of the trial. After obtaining written informed consent from the participants and their legal guardians, participants who meet the eligibility criteria will be randomly assigned to either the treatment or placebo group at a 1:1 ratio. The participants will undergo an 8-week treatment period and an 8-week follow-up.

### Eligibility criteria {10}

#### Diagnostic criteria

A diagnosis of AD will be established according to the Hanifin and Rajka diagnostic criteria. The major criteria include four clinical symptoms (e.g. pruritus, typical morphology, and distribution). The minor criteria included 32 clinical symptoms (e.g. xerosis, ichthyosis, palmar hyperlinearity, and keratosis pilaris). Participants will be diagnosed with AD if they meet three or more major criteria and three or more minor criteria simultaneously.

#### Inclusion criteria

1. Clinical diagnosis of AD
2. Clinical diagnosis for TCM syndromes of damp-heat disturbing heart-mind
3. Scoring Atopic Dermatitis (SCORAD) score between 0 and 50
4. Age 6–12 years
5. Those who volunteered to participate in clinical trial observation and signed the informed consent form
6. Those compliant with treatment completion and follow-up
7. No intake of concomitant drugs during the observation period, except for those specified

#### Exclusion criteria

Participants with any of the following conditions will be excluded:

1. Patients receiving antihistamine drugs, topical corticosteroids, or calcineurin inhibitors in the preceding 1 week
2. Patients with the presence of abnormal liver and kidney function or severe systemic diseases
3. Patients with other skin diseases and bacterial (fungal) infection that could interfere with the assessment of AD severity
4. Patients with poor compliance with TCM treatment
5. Patients with an allergy to the ingredients in the Longmu Tang granule
6. Patients currently participating in another clinical trial

#### Drop out criteria

1. Patients who voluntarily withdraw from the trial because of poor efficacy, adverse reactions, etc.
2. Patients who are lost to follow-up
3. Patients who use prohibited drugs in the protocol

#### Public and patient involvement

Two AD patients in Dr. Na Lang’s clinic have provided an idea for us to place posters in the hospital areas (e.g. the outpatient hall) to recruit patients prior to the trial. The patients will not be involved in other parts of the trial design, such as the recruitment or conduct of this study.

#### Who will take informed consent? {26a}

The researchers will design the questionnaire, recruit patients, and conduct the study. The participants and their guardians will complete the informed consent form if they would like to participate in the study. The informed consent form will explain the purpose, procedure, potential benefits, and risks of the trial.

#### Additional consent provisions for collection and use of participant data and biological specimens {26b}

Prior to obtaining informed consent, we will confirm with the participants the right to use their data, which indicates that the participants’ data will be shared with the other relevant authorities. The trial will not involve collection and use of biological specimens.

### Interventions

#### Explanation for the choice of comparators {6b}

The Longmu Tang placebo granule was selected as the comparator to assess the effects and safety of Longmu Tang granule and to minimise bias.

#### Intervention description {11a}

In the Longmu Tang granule group, participants will be allocated to receive the Longmu Tang granule (9.8 g/sachet) orally with 50 mL of warm water twice a day for 8 weeks. Active granules, each containing 9.8 g of Longmu Tang, are equivalent to 126 g of crude medicine. The placebo granule is composed of 5% crude Longmu Tang and 95% starch with a similar appearance and smell to the Longmu Tang granule. Patients in the control group will take the placebo orally at the same time as those in the Longmu Tang granule group. Both Longmu Tang granule and placebo granules with similar labels and packaging will be provided by Beijing Tcmages Pharmaceutical Co., Ltd. Based on the quality control analysis, the Longmu Tang granule used in the trial should meet the Chinese Medicine Standards of the State Food and Drug Administration.

#### Criteria for discontinuing or modifying allocated interventions {11b}

Participants who fail to complete the study for various reasons or use prohibited drugs in the protocol, regardless of the time or reason, will be allowed to discontinue the study. Serious adverse events will be reported according to standard reporting requirements. The last recorded data for these participants will be included in the data analyses. Data obtained from participants who used prohibited drugs in the protocol will be treated as invalid. For participants who discontinue the trial, the researcher will record in detail the time and reason for discontinuation in the case report form (CRF).

#### Strategies to improve adherence to interventions {11c}

The date and quantity of medicine distribution and return will be recorded. Participants will be required to return both the finished medicine package and unused medicine. Furthermore, providing free health education consultations and daily care for participants will improve adherence to interventions.

#### Relevant concomitant care permitted or prohibited during the trial {11d}

Participants will be discouraged from receiving any other treatment or medication during the study period to prevent any effect on the results. During treatment, if there is substantial exudate or severe itching, the patient will be allowed to moisten the exudate area with the Pifukang solution (specification 50 mL/bottle, national medicine quasi-word Z19990045; Beijing Huayang Kuilong Pharmaceutical Co., Ltd.) 2–4 times a day. An eight-layer medical gauze will be dipped into the diluted lotion, which has been diluted six times with cooled boiled water at 4–10°, gently wrung out, and then applied to the affected skin. Loratadine tablets (Caretan, specification 10 mg × 6 tablets, national medicine quasi-word H10970410; Bayer Medicine (Shanghai) Co., Ltd.) will also be allowed to be taken orally once a day, 10 mg each time, if itching is difficult to resist. Details of the prescriptions and medications used will be recorded in the CRF. The proportion of participants using rescue drugs will be compared between the two groups. The use of other TCMs is prohibited.

#### Provisions for post-trial care {30}

If an adverse event occurs during the clinical trial, the committee of medical experts will determine whether it is related to the Longmu Tang granule. Xiyuan Hospital of China Academy of Chinese Medical Sciences will cover the cost of treatment and the corresponding economic compensation for the damage related to the trial.

### Outcomes {12}

#### Primary outcome

The primary outcome of the trial is the efficacy of the Longmu Tang granule in improving the clinical symptoms of patients with AD after 8 weeks of treatment. The main evaluation of the primary outcome will be the change in the SCORAD score [18] from baseline to the end of the 8-week treatment. SCORAD was proposed by the European Task Force on AD. SCORAD includes the area (A) and severity (B) of skin lesions and the subjective symptoms of pruritus and their effects on sleep (C). The *SCORAD* system is internationally recognised as the gold standard for AD scoring. The SCORAD score ranges from 0 (best) to 103 (worst), with a minimal clinically important difference (MCID) of 8.7 [19]. Higher scores indicate more severe symptoms. The SCORAD scores of the participants before and after treatment will be statistically analysed.

#### Secondary outcomes

1. The change in the Children's Dermatology Life Quality Index (CDLQI) score [20] (from baseline to week 8): the CDLQI is the most commonly used life index score of dermatological quality. This study will recruit children aged 6–12 years, so the CDLQI score will be used. The total CDLQI score ranges from 0 (best) to 30 (worst), with 6 as the MCID [21].
2. The change in the number cancellation test (NCT) score (from baseline to week 8): the NCT is a traditional method used to evaluate attention function; the NCT score will be measured before and after the treatment, respectively, for 3 minutes each time, including the NCT net score and cancellation error rate, number of times of correct cancellation (the greater the better), number of times of incorrect cancellation, and number of times of omitted cancellation (the less the better), which will be compared before and after treatment. Raw score = the number of times of correct cancellation; net score = raw score – (the number of times of incorrect cancellation + 1/2 of the number of times of omitted cancellation); error rate = (the number of times of incorrect cancellation + 1/2 of the number of times of omitted cancellation) / raw score × 100%.
3. Participants’ expectations of the Longmu Tang granule will be assessed at baseline using the following two questions: “Do you think the Longmu Tang granule will be effective for treating the illness?” and “Do you think the Longmu Tang granule will be effective for relieving the related symptoms of AD?” The response options will be ‘yes’, ‘no’, or ‘unclear’.
4. Integral TCM syndrome score (from baseline to week 8 and 12; the scores and details are presented in Tables 2 and 3). A scale of 0–6 points is used to score patients with AD according to the severity of clinical symptoms.
  Significantly effective: inhibition or complete suppression of primary and secondary clinical symptoms and reduction of the syndrome score by ≥ 95%
  Effective: significant improvement in clinical symptoms and reduction in the syndrome score by 70–95%
  Ineffective: improvement in clinical symptoms and reduction in the syndrome score by 50–70%
  Invalid: reduction of treatment syndrome score by < 50%
5. Long-term control: the evaluation of long-term control will be based on the change in the SCORAD score and change in the CDLQI score from baseline to the end of the 8-week follow-up after treatment.

**Table 2:** Traditional Chinese medicine syndrome score

	None	Mild		Moderate		Severe	
Primary symptoms							
Itch	0	2		4		6	
Patterns of skin lesion	0	2		4		6	
Area of skin lesion	0	< 10%	10–29%	30–49%	50–69%	70–89%	90–100%
	0	1	2	3	4	5	6
Secondary symptoms							
Restlessness	0	1		2		3	
Dry mouth	0	1		2		3	
Sleeplessness	0	1		2		3	
Dry stool	0	1		2		3	
Scanty dark urine	0	1		2		3

**Table 3:** Detailed description of symptoms according to Traditional Chinese medicine

	None	Mild	Moderate	Severe
Itch	No symptom	Occasional occurrence and no influence on study and life	Regular occurrence and have influence on study and life	Frequent occurrence and have serious influence on study and life
Patterns of skin lesion	No symptom	Erythema, papules, or blisters	Oozing	Skin thickening (lichenification)
Area of skin lesion	Calculated based on PASI score			
Restlessness	No symptom	Slight	Heavy	Heavy; irritable
Dry mouth	No symptom	Slight; no need to drink water	Severe; need to drink water occasionally	Intolerable; need to drink water frequently
Sleeplessness	No symptom	Slower falling asleep or occasional waking	Difficult to fall asleep and wake up easily	Hardly able to sleep
Dry stool	No symptom	Dry stool; once per day	Dry stool, 1 time in 2–3 days	Difficult stool, > 3 days each time
Scanty dark urine	No symptom	Urine volume is fine, slightly yellowish in colour	Small amount of urine, yellow colour, slightly hot	Scanty dark urine

*Abbreviation*: *PASI* Psoriasis Area Severity Index

#### Exploratory outcomes

Serum inflammatory cytokines, such as serum immunoglobulin E, tumour necrosis factor-α, interleukin-1, and interleukin-6, will be detected using an enzyme-linked immunosorbent assay (ELISA) at baseline and week 8. ELISA kits have been purchased from Sigma-Aldrich.

#### Safety measures

Safety measures include laboratory indices (routine blood and urine test results, and kidney and liver function test results) and adverse events. All adverse events will be tracked for 16 weeks after randomisation.

#### Safety assessment

Full blood count and urine, kidney, and liver function tests will be performed before and after treatment. All details of severe adverse events (SAEs) and adverse events (AEs) will be recorded in the CRF. Any serious adverse events will be reported to the Ethics Approval Committee of Xiyuan Hospital of China Academy of Chinese Medical Sciences within 24 h. Safety will be monitored at every visit for up to 8 weeks after the end of the intervention.

#### Participant timeline {13}

**Fig. 1:**
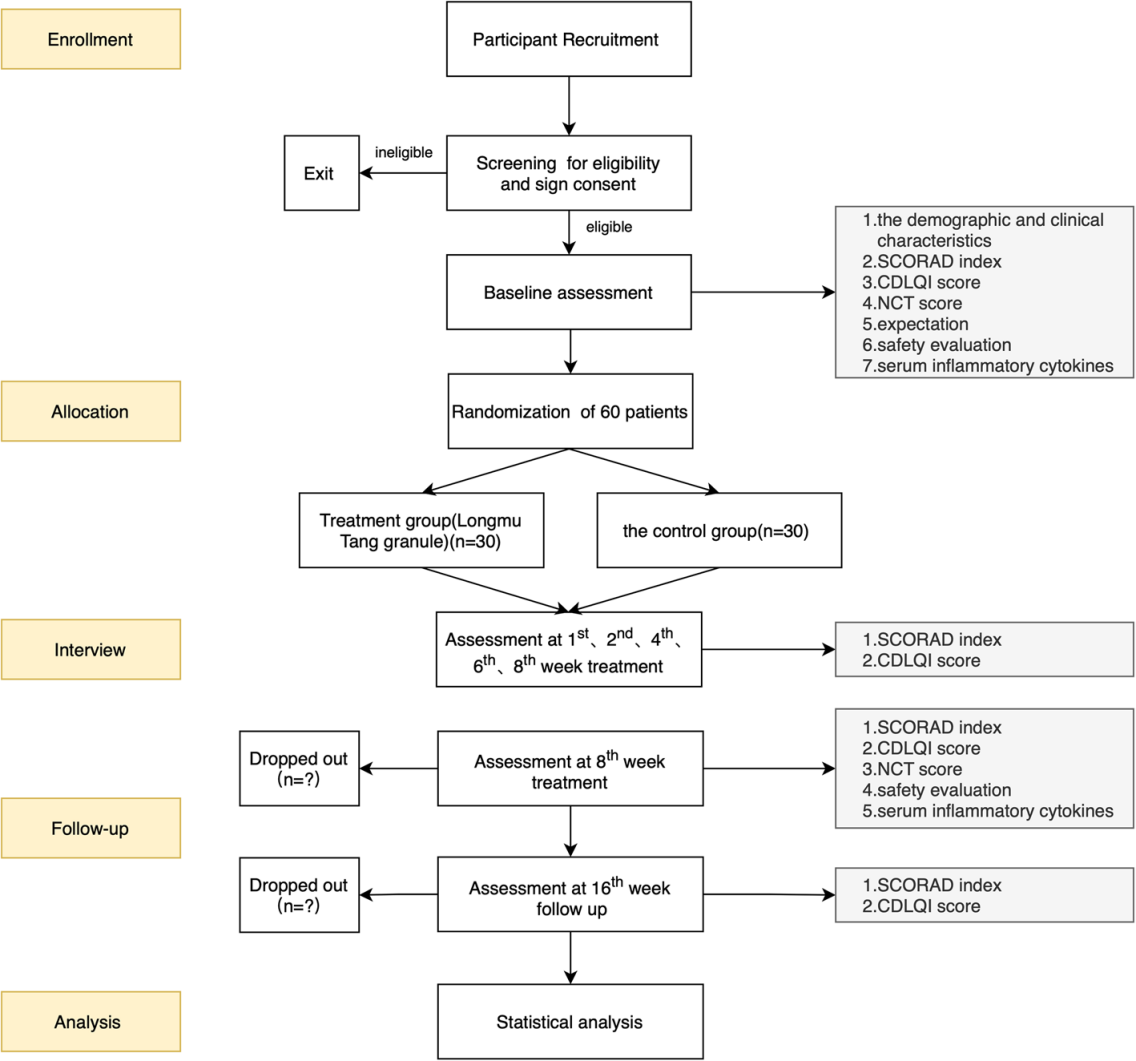
An outline of the procedures

The visit schedule for all assessments (baseline, primary outcome, secondary outcomes, exploration outcomes, and security indicators) is shown in Fig. 1. At each assessment timepoint, the same investigator will evaluate the participants. During the treatment period (i.e. from baseline to week 8), all combined therapeutic medicines will be recorded, including their name, dose, and course. Participants’ expectations of the Longmu Tang granule will be assessed at baseline. The primary outcome and CDLQI score will be evaluated at baseline and at the first, second, fourth, sixth, and last visits. Exploratory outcomes, NCT scores, and safety indicators will only be evaluated at baseline and at the last visit. Table 4 summarises the schedule of screening, enrolment, intervention, assessments, and data collection.

**Table 4:**
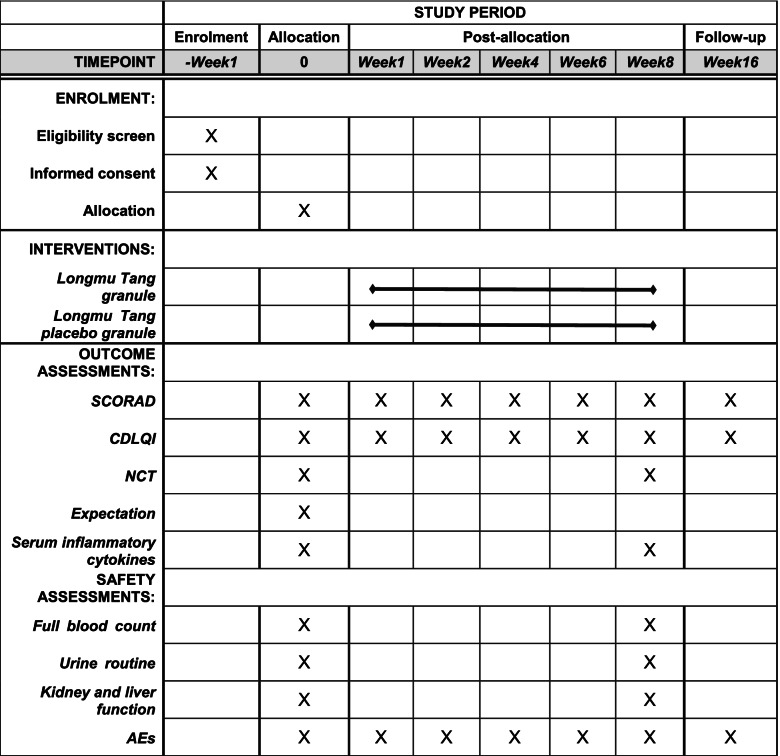
Schedule of enrolment, interventions, and assessments

*Abbreviations*: *SCORAD* Scoring Atopic Dermatitis, *CDLQI* Children’s Dermatology Quality Life Index, *NCT* number cancellation test, *AEs* adverse events

#### Sample size {14}

The sample size was calculated based on the primary outcomes, which were the change in the SCORAD score from baseline to the end of week 8. In our pilot trial [17], with 20 patients in the Longmu Tang group and 20 patients in the control group, the mean (± standard deviation) reductions in the SCORAD score after 8-week treatment were 20.76 ± 8.05 and 6.35 ± 8.69 in the Longmu Tang and control groups, respectively. In a previous study [22], the mean reduction in the SCORAD score in the Longmu Tang group was 15, with 31 patients in the Longmu Tang group and 31 patients in the control group. In another previous study [23], the mean reduction in the SCORAD score in the Longmu Tang group was 12.86, with 62 patients in the Longmu Tang group and 60 patients in the control group. Considering the three trials and number of patients in each of the three trials, we estimated the mean (± standard deviation) reduction in the SCORAD score after 8-week treatment in the experimental and control groups to be 15 ± 8.69 and 6.35 ± 8.69, respectively. Assuming an alpha risk of 5% and beta risk of 10% using the GPOWER software (version 3.1.9.7), a sample size of 46 (23 participants in each group) was calculated. Considering a 20% loss to follow-up, this trial will require 60 participants (30 participants in each group).

#### Recruitment {15}

Sixty participants will be recruited from March 2021 to March 2022 at Xiyuan Hospital of China Academy of Chinese Medical Sciences via Internet advertisements (using WeChat) and posters in the hospital areas (e.g. the outpatient hall). Dermatologists will also ask the patients if they would like to participate in this trial during the treatment process of AD. A dermatologist will be responsible for screening potential participants and assessing whether they meet the eligibility criteria. A research assistant will provide the participant and their guardian with an informed consent form and explain it in detail. The informed consent form will cover the purpose, procedure, potential benefits, and risks of the trial. The research assistant will perform a baseline assessment of all participants (− 1 to 0 weeks) before randomisation. The following demographic and clinical characteristics of the participants at baseline will be recorded in the CRF: age, sex, race, birthplace, the body mass index (kg/m^2^), current address, history of allergic diseases, family history, severity of AD assessed by the SCORAD index (mild [0–24 score], moderate [25–50 score], severe [51–103 score]), quality of life assessed by the CDLQI, cognitive ability assessed by the NCT, and safety evaluation assessed by laboratory indices (full blood count and urine, kidney, and liver function test results). An outline of the procedure is illustrated in Fig. 1.

### Assignment of interventions: allocation

#### Sequence generation {16a}

The random code will be generated by an independent statistician from the Good Clinical Practice (GCP) Centre of Xiyuan Hospital of China Academy of Chinese Medical Sciences. Participants will be randomly assigned to either the treatment or placebo group, at a ratio of 1:1 and a fixed block of 4.

#### Concealment mechanism {16b}

The number of randomisation sequences and information regarding group allocation will be placed in ordered envelopes and subsequently sealed to ensure randomisation concealment. The medicines will be labelled with random codes on the small and large packages by GCP specialists of the Xiyuan Hospital of China Academy of Chinese Medical Sciences.

#### Implementation {16c}

All participants and researchers will be blinded to the group assignment until trial completion. The same blinding code generated by the statisticians will be provided to the research institution and will not be broken during the trial period, except in the case of a serious adverse event that requires urgent breaking of the blinding code.

### Assignment of interventions: blinding

#### Who will be blinded {17a}

All participants and researchers will be blinded until the trial is completed.

#### Procedure for unblinding if needed {17b}

An emergency envelope contains randomised codes and group information corresponding to each patient. Once a serious event occurs and patients need to be treated, the project leader will decide to initiate unblinding and open the corresponding emergency envelope. Then the case will be treated as a shredded case.

### Data collection and management

#### Plans for assessment and collection of outcomes {18a}

The assessment and collection of outcomes are described in the outcomes section {12}, and at each visit, data will be recorded in the CRF, which can be provided by the corresponding author.

#### Plans to promote participant retention and complete follow-up {18b}

To maximise compliance and retention throughout the study, researchers will ensure that the study schedule and participation requirements are fully explained before consent is obtained and that participants are aware of the potential risks and benefits of the treatment. Each participant will be provided with a copy of the signed informed consent form, containing the contact information of the investigators, so that they can contact the primary investigators if required. Moreover, all participants will receive phone calls before each visit to remind them of their time. The participants will undergo free examinations and study medicine.

#### Data management {19}

Data will be imported into Epidata software (Doctors Without Borders) by two independent researchers. All researchers will receive professional training about correct assessments, procedures regarding the use of the study medicine, CRF completion, and use of the Epidata before and during this trial. Auditors from the Capital’s Funds for Health Improvement and Research will supervise all trial-related procedures and data management.

#### Confidentiality {27}

All participants’ data will be collected in a specific reception room instead of in public areas to avoid revealing their personal information to others. Initials of patient names will be used on the CRF, and all documents will be stored confidentially and accessible only to members of this study. Personal information about the potential and enrolled participants will only be consulted by the researchers of this study and will not be shared.

#### Plans for collection, laboratory evaluation, and storage of biological specimens for genetic or molecular analysis in this trial/future use {33}

Not applicable. This trial will not involve biological specimen collection for genetic or molecular analysis.

### Statistical methods

#### Statistical methods for primary and secondary outcomes {20a}

Data analysis will be completed using the Statistical Package of Social Sciences software (version 22.0; IBM Corp.), according to the per-protocol and intention-to-treat principles. Normally distributed continuous variables will be reported as mean ± standard deviation, and non-normally distributed continuous variables will be reported as median (interquartile range). The two-independent sample *t*-test, Wilcoxon test, or paired *t*-test will be used to analyse continuous variables, and the chi-square test and Fisher exact probability test will be used to analyse categorical variables. The two-sided test will be used uniformly in the hypothesis testing. A *P-*value < 0.05 will indicate that the difference is statistically significant, and a *P*-value < 0.01 will indicate that the difference is highly statistically significant.

#### Interim analyses {21b}

Not applicable. There were no adverse events in our pilot trial and earlier studies, so oral administration of Longmu Tang granule was deemed to be a low-risk intervention.

#### Methods for additional analyses (e.g. subgroup analyses) {20b}

A subgroup analyses by sex will be performed in the future.

#### Methods in analysis to handle protocol non-adherence and any statistical methods to handle missing data {20c}

Participants with poor adherence and a drug compliance < 80% or those who are no longer receiving medication and undergoing testing during the study will be included in the intention-to-treat analysis and excluded from the per-protocol analysis. A multiple data imputation procedure will be used to impute the missing data.

#### Plans to give access to the full protocol, participant level-data, and statistical code {31c}

The full protocol, participant-level data, and statistical code are available from the corresponding author upon reasonable request.

### Oversight and monitoring

#### Composition of the coordinating centre and trial steering committee {5d}

The coordinating centre is the Department of Dermatology of Xiyuan Hospital of China Academy of Chinese Medical Sciences, which will help recruit participants and obtain their consent. The trial steering committee is Capital’s Funds for Health Improvement and Research, which will supervise the trial and review its progress.

#### Composition of the data monitoring committee, its role and reporting structure {21a}

Not applicable. This trial involves a low-risk intervention.

#### Adverse event reporting and harms {22}

All details of SAEs and AEs will be recorded in the CRF and reported to the primary researcher, including symptoms and severity, timing, action taken, and outcomes. Any serious adverse events will be reported to the Ethics Approval Committee of the Xiyuan Hospital of China Academy of Chinese Medical Sciences within 24 hours. If an adverse event occurs in a clinical trial, the committee of medical experts will determine whether it is related to the experimental medicine and further decide whether to terminate the treatment based on the patient’s condition. Other unintended effects of the study interventions and conduct will also be recorded and reported.

#### Frequency and plans for auditing trial conduct {23}

The trial will be supervised and audited by experts from the Capital’s Funds for Health Improvement and Research and Ethics Approval Committee of the Xiyuan Hospital of China Academy of Chinese Medical Sciences once a year.

#### Plans for communicating important protocol amendments to relevant parties (e.g. trial participants, ethical committees) {25}

We will notify the Ethics Approval Committee of the Xiyuan Hospital of China Academy of Chinese Medical Sciences and Capital’s Funds for Health Improvement and Research when there is any important change in the study process, such as a change in the sample size.

#### Dissemination plans {31a}

The final results will be submitted to Capital’s Funds for Health Improvement and Research in the form of a research report. The trial results will be disseminated via peer-reviewed publications, scientific conferences, and meetings.

## Discussion

AD is a complex chronic inflammatory skin disease that involves the critical components of epidermal barrier disruption, dysregulation of the immune response, and genetic predisposition. AD-related itching and frequent scratching behaviour substantially impair the patient's quality of life in health-related aspects, such as sleep, physical activity, and psychosocial and mental functioning. Roth et al. [24] found that children with AD were more likely to suffer from ADHD than children without AD, and they were more likely to show attention deficits in particular. Thus, the optimal treatment regimen for AD should include anti-inflammatory agents, improvement of pruritus, and attenuation of skin lesions.

The clinical efficacy of TCM in treating AD has been gradually recognised in attenuating skin lesions, preventing recurrence, and reducing itchiness [25]. The use of TCM to treat AD has a long history, and is especially effective in preventing disease recurrence, maintaining long-term remission, and reducing disease burden.

However, many of the reported outcomes from clinical studies of TCM formulas or herbal medicines are not widely accepted because of the lack of strict protocol design to meet internationally accepted standards. To accomplish our trial goal, we proposed a clinical trial design and will follow the protocol to conduct the trial with a rigorous methodology and high-quality control. The Longmu Tang granule is produced using a standardised procedure according to the Good Manufacturing Practice (GMP) protocol, which guarantees homogeneous active components and reliable trial outcomes. Currently, there are few TCM treatments involving syndrome differentiation therapy in patients with mild-to-moderate AD. To our knowledge, this is the first clinical trial to explore the efficacy and safety of the Longmu Tang granule for treating AD associated with damp-heat disturbing heart-mind. Our results from a previous clinical study showed that the Longmu Tang granule can improve the severity of symptoms in AD patients with an efficacy rate of 70% [17]. These results may confer broad therapeutic benefits as a disease syndrome differentiation therapy for AD and itchiness.

There are some limitations to this study. First, the study will be performed at a single centre, so the results may not be able to be extrapolated to other ethnic groups or regions. Second, the sample size of this study is small, which may lead to overestimation of the efficacy of the Longmu Tang granule.

In conclusion, this trial will provide high-quality evidence on the effectiveness and safety of the Longmu Tang granule, which can be used to guide clinical practice for AD.

## Trial status

The protocol version number is V3.2, and the date is 14 May 2020. Recruitment commenced in March 2021 and will end in March 2022.

## Data Availability

The principal investigators will have access to the final trial database, which will be available on reasonable request. Participants’ personal information will be confidentially before, during and after the trial.

## Abbreviations

AD: Atopic dermatitis
TCM: Traditional Chinese medicine
SCORAD: Scoring Atopic Dermatitis
CDLQI: Children’s Dermatology Life Quality Index
NTC: Number cancellation test
ADHD: Attention-deficit/hyperactivity disorder
CDLQI: Children’s Dermatology Life Quality Index
ELISA: Enzyme-linked immunosorbent assay
SAEs: Severe adverse events
AEs: Adverse events
CRF: Case report form
